# Comparing the genomes of cutaneous melanoma tumors to commercially available cell lines

**DOI:** 10.18632/oncotarget.22928

**Published:** 2017-12-04

**Authors:** Stephen A. Luebker, Weiwei Zhang, Scott A. Koepsell

**Affiliations:** ^1^ Department of Pathology and Microbiology, University of Nebraska Medical Center, Omaha, NE

**Keywords:** pre-clinical, tumor model, sequencing

## Abstract

Insulated culture environment and prolonged propagation contribute to known limitations of cell lines, and selection is often limited to availability or favorable growth characteristics. To better characterize and improve selection of cell lines, we compared 60 melanoma cell lines profiled by the Cancer Cell Line Encyclopedia and 472 cutaneous melanoma tumors profiled by The Cancer Genome Atlas by DNA sequence and copy number alterations. All samples were scored for stromal and immune cell composition by the ESTIMATE algorithm, and 412 tumors with ≥ 60% tumor cell fraction were compared to cell lines. Uncharacterized early passage cell lines that lacked *BRAF*, *NRAS*, or *NF1* mutations had near zero mean Pearson correlation of copy number alterations per gene to tumors and also tended to have higher stromal scores. The Comet Exact Test was applied to tumors and cell lines identifying three pairs of genes mutated in a mutually exclusive pattern in tumors but not cell lines: *BRAF* and *NRAS*, *BRAF* and *NF1*, as well as *NRAS* and *PTEN*. Additionally, 31 genes were more frequently mutated in cell lines than tumors. Avoiding cell lines with co-occurring mutually exclusive mutations and the fewest differentially mutated genes within a known distribution of genetic similarity to tumors by copy number alterations may optimize selection.

## INTRODUCTION

Incidence of cutaneous melanoma in the United States occurred at a rate of 26.8 per 100,000 males and 16.6 per 100,000 females from 2009–2013 [1]. Since the 1960′s, incidence of cutaneous melanoma has continued to increase in Caucasian populations North America and Europe with a stable mortality rate, while mortality rates have increased in East Asian populations despite relatively low incidence [2, 3]. Additionally, African American populations with cutaneous melanoma have lower survival rates relative to Caucasians despite having lower rates of occurrence [4]. Cutaneous melanoma persists as a deadly disease if not diagnosed and surgically removed early in its progression with a 97% 5-year survival rate for stage IA, but survival drops dramatically once metastases have spread to regional lymph nodes with only 78% 5-year survival rate for stage IIIA and 15–20% 5-year survival rate for stage IV [5]. Targeted therapies exist for treating cutaneous melanoma at more advanced stages including RAF and MEK inhibitors as well as immune blockade therapy through the PD1 antibody treatment, but primary and acquired therapy resistance both limit increases in patient survival [6, 7].

Cancer cell lines continue as a pre-clinical tool for development of novel therapeutics and diagnostics. Cell lines have been widely used for drug screening and mechanistic studies in 2D and 3D cell cultures as well as xenograft models, but immortalized cancer cell lines limit the generalizability of conclusions due to clonal homogeneity and the lack of a full complement of stromal and immune cell types [8]. Characterization and evaluation of the similarity of cancer cell lines to tumors *in vivo* by their molecular characteristics makes a first step to increasing translational efficiency of pre-clinical studies. Significant genetic alterations can be applied as a basic metric before investigating cancer biology with cell lines including significant mutations of oncogenes or tumor suppressors, karyotype similarity, and DNA methylation. The number of cell line passages before analysis also critically influences results since genomic features may change during propagation and maintenance of cell lines [9]. Recently, comparative studies have been carried out to evaluate the overall genomic, transcriptomic, and to a limited extent proteomic similarity between cell lines and tumors from multiple cancer types [10–14]. These studies utilize publicly available multi-platform biological data resources and sharing platforms including but not limited to The Cancer Genome Atlas (TCGA), The Gene Expression Omnibus, The Cancer Cell Line Encyclopedia (CCLE), and the COSMIC Cell Lines Project [15–18].

The original TCGA study that characterized cutaneous melanoma (TCGA-SKCM) included 67 primary tumors and 266 metastatic tumors, and as samples continue to be analyzed these numbers have increased to 104 primary tumors and 367 metastatic tumors included in this study [19]. Commercially available cell lines characterized by CCLE derive from many tumor types including 60 melanoma cell lines [16]. We hypothesized that comparison of melanoma cell lines to tumors primarily by their DNA sequence and DNA copy number alterations provides the most stable and broadly applicable evaluation of similarity. Vincent & Postovit previously ranked similarity of cutaneous melanoma cell lines to tumors from 19 patients by averaging all correlation coefficients of mRNA expression between cell lines and 1,246 individual melanoma cells profiled by single cell RNA-seq [13]. Though a direct tumor cell to tumor cell comparison revealed important differences in the types of genes expressed between cell lines and tumors, RNA expression patterns are dynamic and change in response to environmental cues like stress from hypoxia and inflammation to name two common examples [20, 21]. Under normal culture conditions, cell lines are not receiving stromal cues; therefore, RNA expression may not be the most accurate representation of cell line similarity to tumor cells *in vivo*. To address this limitation, we focus on genomic data, particularly DNA mutations and DNA copy number alterations, from 470 cases included in The Cancer Genome Atlas Skin Cutaneous Melanoma (TCGA-SKCM) study and 60 melanoma cell lines profiled by CCLE.

Aran et al. applied multiple molecular data analysis approaches to estimate the tumor cell fraction of all tumor samples profiled by TCGA and found highly variable purity, particularly in TCGA-SKCM samples, which confounds conclusions drawn from next generation sequencing data [22]. To address this limitation of the TCGA-SKCM data, comparative analyses in this study includes only TCGA-SKCM samples with a tumor cell fraction ≥ 60% calculated from scores derived from the ESTIMATE algorithm [23]. Cutaneous melanoma cell lines were evaluated by (1) the presence of significantly mutated genes defined by TCGA-SKCM, (2) the number of differentially mutated genes, (3) the co-occurrence of mutations in cell lines that were found to be mutually exclusive in TCGA-SKCM, and (4) the correlation of copy number alterations per gene in focally amplified and deleted regions identified by GISTIC 2.0 analysis. Gross genomic features were evaluated by the difference in mutational burden between tumors and cell lines and the amount of copy number alteration across the entire genome.

## RESULTS

### Data summary

**Figure 1:**
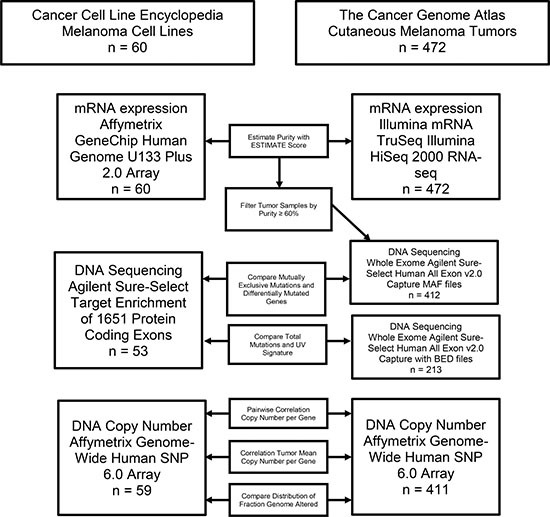
Flow chart outlining the analysis pipeline and samples included in each comparison

The CCLE study included 62 cell lines annotated as being skin derived, and 60 of those are defined as cutaneous melanoma cell lines. BJHTERT, an immortalized fibroblast cell line, and GRM, a likely pancreatic cancer cell line by SNP identity according to CCLE annotation, were excluded from comparative analysis. TCGA-SKCM profiled 472 fresh frozen cutaneous melanoma tumor samples from 470 cases. Target paired-end sequencing was performed on CCLE samples using Agilent Sure-Select Target Enrichment System including 1651 selected genes [16]. Whole exome paired-end sequencing was performed on TCGA-SKCM samples using Agilent Sure-Select Human All Exon v2.0 capture [19]. Copy number estimation for both TCGA-SKCM and CCLE samples were profiled with Affymetrix Genome-Wide Human SNP 6.0 Array and segmented with the Circular Binary Segmentation algorithm then normalized as ploidy corrected log2 ratios [16, 19]. CCLE profiled mRNA expression using an Affymetrix GeneChip Human Genome U133 Plus 2.0 Array and converted probe intensities to gene-wise expression with Robust Multi-array average and quantile normalization [24]. TCGA-SKCM profiled mRNA expression using Illumina paired-end RNA sequencing and used RSEM software for normalization [25]. Every data type was not available for every sample. Therefore, each analysis was limited to samples with the data available (Figure 1).

### Tumor and cell line ESTIMATE scores

**Figure 2:**
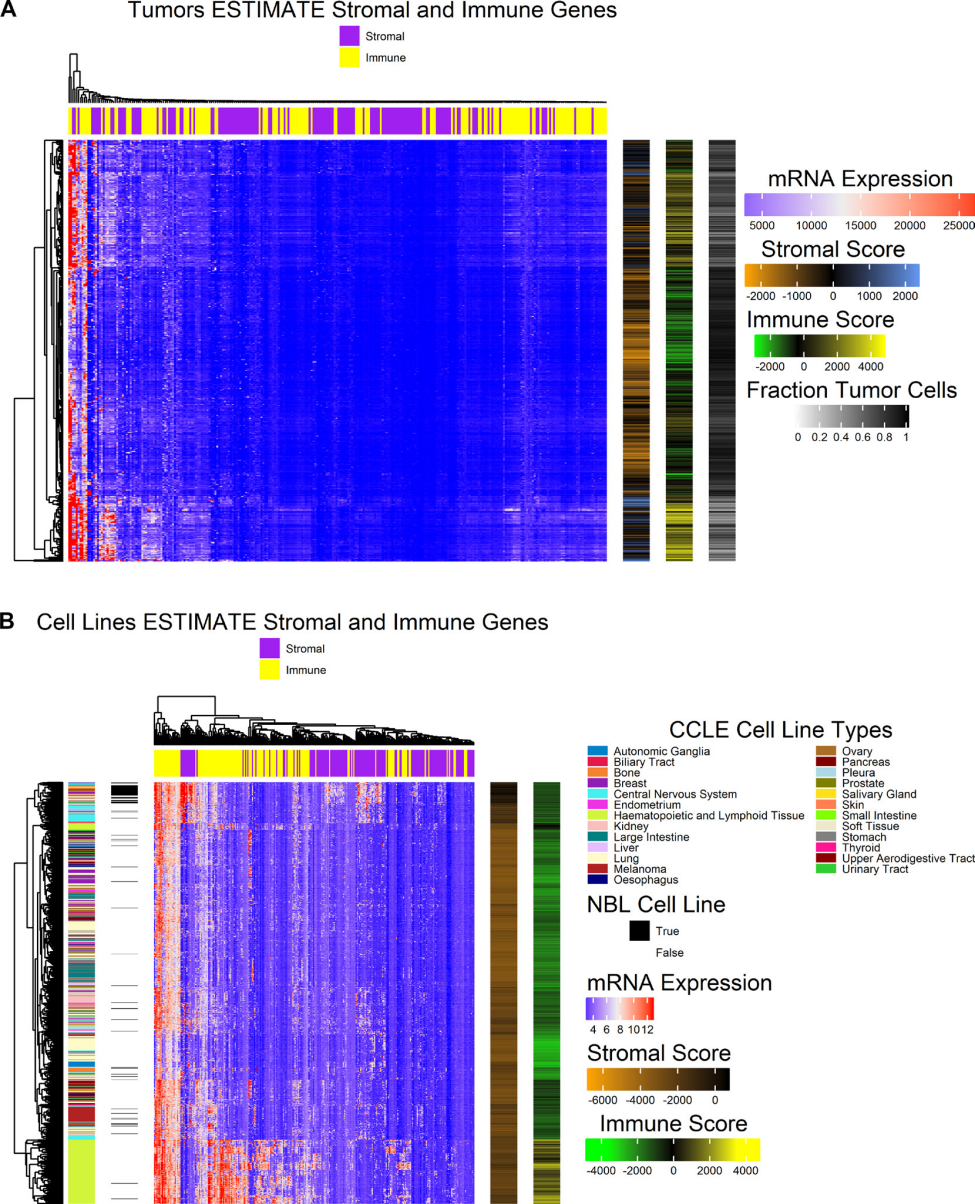
Heat maps illustrate the normalized expression values for genes included in the ESTIMATE algorithm for stromal and immune gene signatures (**A**) Heatmap (center) depicting hierarchically clustered normalized expression values for stromal and immune ESTIMATE gene sets (columns) for tumor samples (rows). Clustering was carried out with Euclidean distance and Ward’s agglomeration method. Annotations (right) include stromal score, immune score, and the fraction tumor cells estimated. (**B**) Heatmap (center) depicting hierarchically clustered normalized expression values for stromal and immune ESTIMATE gene sets (columns) for all cell lines profiled by CCLE (rows). Clustering was carried out with Euclidean distance and Ward’s agglomeration method. Sample annotations (left) include cell line type and whether or not the cell line was originally sourced from the Naval Biosciences Laboratory (NBL). ESTIMATE algorithm results (right) illustrate stromal score and immune score.

In order to control for variability in tumor sample composition, the tumor cell fraction was estimated for cell lines using mRNA microarray expression data and for tumors using RNA-seq expression data with the ESTIMATE algorithm [23]. The TCGA-SKCM study required tissue sample composition ≥ 60% tumor nuclei with ≤ 20% necrosis by histological review with further macrodissection if the criteria were not met for DNA/RNA extraction [19]. To meet this minimal tumor fraction threshold, the TCGA-SKCM tumor data set was filtered to only include samples with tumor cell fraction ≥ 60% calculated from the ESTIMATE score as shown in Figure 1. In the normalized expression data for tumors, 139 of 141 genes were common with the ESTIMATE stromal gene set and 141 of 141 immune genes were common with the ESTIMATE immune gene set. Hierarchical clustering of normalized mRNA expression of the two gene sets is shown in Figure 2A. Two tumor samples form a cluster with low estimated tumor purity and high expression of both immune and stromal genes. A sub-cluster of tumors with low estimated tumor cell fraction show strong expression of immune genes.

ESTIMATE scores were also calculated for all cell lines included in the CCLE study. In the GCT file of normalized microarray expression data, 139 of the 141 genes were common with each the ESTIMATE stromal gene set and immune gene set respectively. Stromal and immune scores across cell lines are very low relative to those found in melanoma tumors, and clusters tend to form by the derived cell type (Figure 2B). Hematopoietic and lymphoid tissue cell lines form a cluster characterized by higher expression of immune genes and higher immune scores relative to other cell lines as expected by their cell type. A sub-cluster of the non-hematopoietic or lymphoid derived cell lines shows high expression of stromal genes including central nervous system cell lines and multiple early passage primary cell cultures made available by ATCC (https://www.atcc.org/) with variable mixtures of tumor and stromal cells that were originally provided by the Naval Biosciences Laboratory (NBL). HS600T, HS834T, HS688AT, HS839T, HS934T, and HS940T are melanoma derived cell cultures made available by ATCC as uncharacterized early passage lines, and all are included in the cluster with the highest stromal score. Additionally, BJHTERT, an immortalized fibroblast cell line, and HS895T, a fully characterized cell line, both cluster with the higher stromal scoring cell lines. Many other cell lines originally sourced by NBL were further developed to fully characterized cell lines and generally cluster according to their cell type.

### Comparing DNA copy number

**Figure 3:**
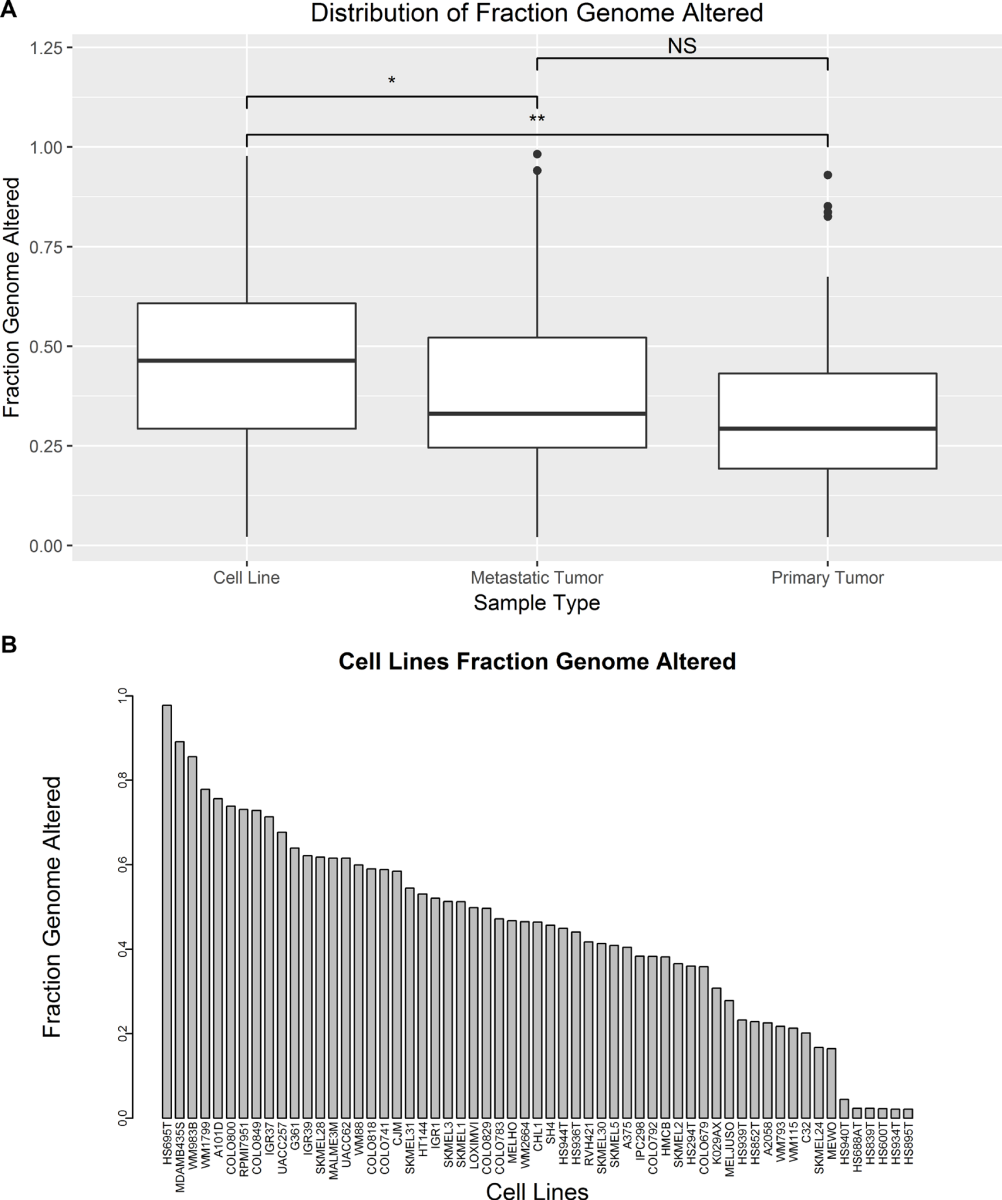
Comparison of FGA between tumors and cell lines (**A**) Boxplots compare the distribution of FGA between cell lines, metastatic tumors, and primary tumors (^*^p-value < 0.05, ^**^p-value < 0.01 by ANOVA and Tukey’s Method). (**B**) Barplot compares FGA of each cell line.

Segmented copy number alterations were compared between cell lines and tumors by their fraction genome altered (FGA). FGA was applied as described by Domcke et al. to measure of the proportion of copy number segments that are either amplified or deleted above a chosen threshold as described in the methods section [10]. Mean FGA was found to be significantly different between cell lines (*n* = 59), metastatic tumors (*n* = 310), and primary tumors (*n* = 101) (*p* < 0.05 one-way ANOVA). Cell line mean FGA (0.45 ± 0.23) was significantly higher than both primary tumors (0.33 ± 0.19) and metastatic tumors (0.38 ± 0.20) (*p* < 0.05 Tukey’s Method). Mean FGA was not significantly different between primary and metastatic tumors (*p* = 0.09 Tukey’s Method) (Figure 3A). HS695T, MDAMB435S, and WM983B are the top three cell lines by FGA with more than half of all copy number segments altered (Figure 3B). HS940T, HS688AT, HS839T, HS600T, HS934T, and HS895T have the lowest FGA of the melanoma cell lines. The mean FGA for all tumors combined is 0.37 ± 0.20 (min = 0.02, max = 0.98).

**Figure 4:**
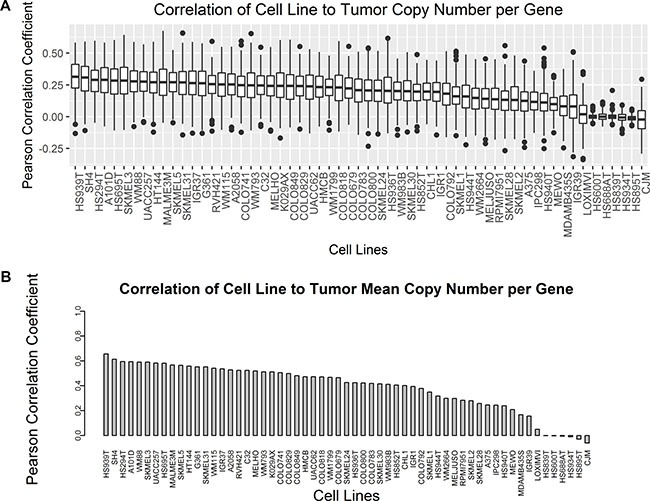
Pearson correlation coefficients of copy number per gene were compared between cell lines and tumors for all genes (**A**) Boxplots compare the distribution of all pairwise Pearson r values between each cell line and each tumor. (**B**) The average copy number per gene was calculated across all tumors, and Pearson r values were calculated for each cell line relative to the tumor mean value represented as a barplot.

In order to compare overall copy number similarity between cell lines and tumors across all gene coding regions, the average copy number per gene was calculated as described in the methods section. Sample Pearson correlation coefficients were calculated for the copy number alteration per gene between each cell line and each tumor sample to determine the distribution of similarity. Comparison of copy number across all genes between individual cell lines and tumors results in a broad range of Pearson r values with multiple outliers across cell lines (Figure 4A). HS939T (mean = 0.31 ± 0.13) and SH4 (mean = 0.31 ± 0.14) both had the highest mean Pearson r value with tumors of all the cell lines. Interestingly HS939T, an uncharacterized early passage melanoma cell line, had the highest mean Pearson r value, but other uncharacterized early passage lines including HS600T, HS688AT, HS839T, HS934T, and HS895T all had mean Pearson r values near zero. These same five uncharacterized cell lines also had FGA near zero possibly accounting for the lack of a linear association with tumor sample copy number per gene. Two fully characterized cell lines, CJM and LOXIMVI, also have mean Pearson r values near zero, but these two cell lines have larger standard deviation relative to the five uncharacterized early passage cells with near zero correlation with tumor samples. Sample Pearson correlation coefficients were also calculated for each cell line relative to the average copy number per gene across all tumors (Figure 4B). HS939T and SH4 are also the top two cell lines by correlation to the tumor mean copy number per gene. The uncharacterized early passage cell lines as well as LOXIMVI and CJM also have very low correlation to the tumor mean copy number per gene. The average Pearson r value of all pairwise comparisons and the correlation to the tumor mean tend to maintain the same order when ranked from highest to lowest, but correlation to the tumor mean resulted in much larger Pearson r values.

**Figure 5:**
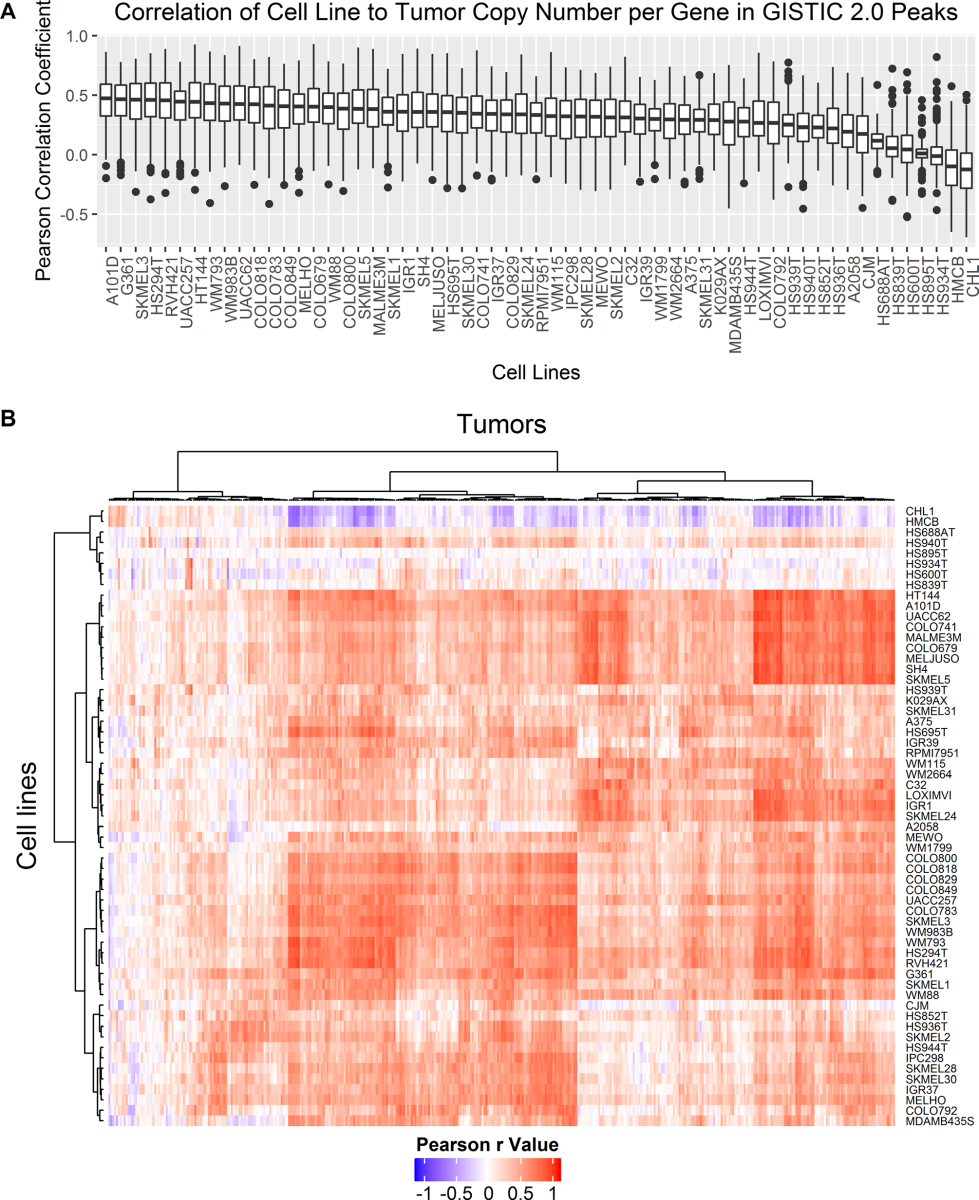
Pearson correlation coefficients of copy number per gene were compared between cell lines and tumors for genes falling within GISTIC 2.0 peak focal amplifications and deletions detected in metastatic tumor samples (**A**) Boxplots compare the distribution of all pairwise Pearson r values between each cell line and each tumor for genes found in focal amplifications or deletions by GISTIC 2.0 analysis. (**B**) Hierarchical clustering of all pairwise Pearson r values between each cell line (rows) and each tumor (columns) for genes found in focal amplifications or deletions by GISTIC 2.0 analysis were plotted as a heatmap. Clustering was carried out with Euclidean distance and Ward’s agglomeration method.

In general, Pearson correlation between cell lines and tumors based on copy number per gene was fair to poor with the highest mean value of 0.31. Melanoma tumor samples are a very heterogeneous in terms of copy number alterations with a large range of FGA from 0.02 to 0.98. To narrow the comparison to regions of significance, Pearson r values were calculated between cell lines and tumors by copy number per gene for a subset of genes within the peak of significant focal amplifications and deletions in metastatic melanoma tumors from TCGA-SKCM (*n* = 367) by GISTIC 2.0 analysis provided by the Broad Institute Genomic Data Analysis Center Firehose [26]. Correlation of copy number per gene within significant focal amplifications and deletions presents a more specific metric of similarity between cell lines and tumors as opposed to comparing correlation of CNAs across all genes. The distribution of Pearson r values is different when only considering focal deletions and amplifications rather than comparing CNAs across all genes (Figure 5A). The average difference in the mean Pearson r value across all melanoma cell lines was seventeen times higher for correlation between genes found within focal amplifications and deletions relative to the mean correlation coefficients for CNAs in all coding genes. The mean Pearson r value was higher for comparison of focal amplifications and deletions than across all genes for all samples except CHL1, HMCB, A2058, and HS939T for which the mean Pearson r value decreased. Hierarchical clustering of Pearson r values between each cell line and tumor pairwise comparison of CNAs in genes found in focal amplifications and deletions are shown in Figure 5B. The uncharacterized early passage cell lines HS839T, HS600T, HS934T, HS895T, HS940T, and HS688AT as well as CHL1 and HMCB, two commonly derived cell lines with high SNP identity, form a cluster with poor correlation across all tumor samples. Tumor samples form two main clusters, one with moderate to high correlation to most cell lines and another with low correlation across most cell lines. There was no statistical difference in these two clusters of tumors by primary or metastatic status, tumor stage, or tumor purity (data not shown).

### Comparing mutations

After filtering the TCGA-SKCM MAF file to include the 1,651 genes targeted in the CCLE hybrid-capture sequencing, there were 1,192 genes common between the melanoma cell line (*n* = 53) and melanoma tumor (*n* = 412) MAF files. Single nucleotide polymorphisms, insertions, and deletions were included in the comparison, but synonymous variants were excluded. There were 31 genes that were mutated in significantly higher proportion of cell lines relative to the proportion of tumors (Bonferroni adjusted *p*-value ≤ 0.05 by Fisher’s Exact Test) (Table 1). Eight of the fifteen significantly mutated genes identified in the original TCGA-SKCM study were also mutated in melanoma cell lines including *BRAF*, *TP53*, *NF1*, *NRAS*, *PTEN*, *MAP2K1*, *IDH1*, and *RB1*. These eight genes were tested for mutual exclusivity within each data set with the Comet Exact Test [27]. Single nucleotide polymorphisms, insertions, and deletions were included as criteria for a mutated gene, but synonymous variants were excluded from comparison. There were three pairs of genes that were mutated in a mutually exclusive pattern in tumor samples (*n* = 412): *BRAF* and *NRAS* mutations (Bonferroni adjusted *p*-value < 0.05), *BRAF* and *NF1* mutations (Bonferroni adjusted *p*-value < 0.05), and *NRAS* and *PTEN* mutations (Bonferroni adjusted *p*-value < 0.05) (Table 2). None of the eight genes had a mutually exclusive mutation pattern in cell lines (*n* = 53) (Bonferroni adjusted *p*-value ≥ 0.05) (Table 3). WM88, HS695T, and LOXIMVI cell lines have co-occurring *BRAF* and *NF1* mutations. HS936T and SKMEL30 cell lines have co-occurring *BRAF* and *NRAS* mutations. HS944T has co-occurring *NRAS* and *PTEN* mutations.

**Table 1:** Differentially mutated genes between tumors (*n* = 412) and cell lines (*n* = 53) by Fisher’s Exact Test with multiple testing corrections by the Bonferroni method

Hugo Symbol	Frequency in Cell Lines	Frequency in Tumors	*p*-value
*MAP3K14*	41	0	2.82E-45
*MYST4*	14	0	1.38E-11
*MLL3*	13	0	1.56E-10
*KIAA1409*	8	0	2.07E-05
*ODZ1*	7	0	0.000206
*MYST3*	5	0	0.019293
*SGK269*	5	0	0.019293
*NEK3*	36	1	1.92E-36
*GRIA3*	52	29	1.46E-43
*NR1H2*	46	6	3.69E-46
*MAP3K1*	44	5	8.44E-44
*CLTCL1*	48	14	1.18E-43
*VEGFC*	50	25	2.43E-41
*MAML3*	47	12	3.15E-43
*AKAP12*	44	8	3.12E-41
*PRKDC*	47	34	1.54E-32
*AAK1*	28	7	6.95E-21
*RECQL4*	32	11	4.38E-23
*ITPR2*	37	18	1.56E-25
*CTBP2*	26	8	4.69E-18
*MSH3*	19	7	2.02E-11
*CREB3L2*	24	13	1.52E-13
*ASPH*	16	8	3.86E-08
*MAML2*	17	9	1.27E-08
*CHD1*	22	15	6.93E-11
*PIK3C2G*	34	63	2.46E-10
*AKAP9*	28	44	8.43E-09
*GPR112*	36	84	7.52E-09
*ALPK2*	31	66	1.52E-07
*NCOA3*	18	28	0.000205
*PDE4DIP*	26	53	7.08E-06

**Table 2:** Testing for mutually exclusive mutation patterns found in melanoma tumors (*n* = 412) by the Comet Exact Test with multiple testing correction by the Bonferroni method

Gene 1	Gene 2	NeitherMutated	Gene 2Mutated	Gene 1Mutated	BothMutated	*p*-value
*BRAF*	*NRAS*	98	110	196	8	2.49E-30
*BRAF*	*NF1*	160	48	190	14	4.34E-05
*NRAS*	*PTEN*	259	35	116	2	0.004565
*NF1*	*MAP2K1*	328	22	62	0	0.349177
*BRAF*	*IDH1*	194	14	198	6	1
*BRAF*	*RB1*	198	10	199	5	1
*NF1*	*PTEN*	316	34	59	3	1
*PTEN*	*RB1*	360	15	37	0	1
*NRAS*	*NF1*	246	48	104	14	1
*NRAS*	*MAP2K1*	276	18	114	4	1
*PTEN*	*MAP2K1*	354	21	36	1	1
*BRAF*	*TP53*	175	33	173	31	1
*MAP2K1*	*IDH1*	371	19	21	1	1
*NF1*	*IDH1*	333	17	59	3	1
*PTEN*	*IDH1*	357	18	35	2	1
*MAP2K1*	*RB1*	376	14	21	1	1
*TP53*	*RB1*	336	12	61	3	1
*NF1*	*RB1*	338	12	59	3	1
*TP53*	*IDH1*	332	16	60	4	1
*TP53*	*MAP2K1*	331	17	59	5	1
*NRAS*	*RB1*	285	9	112	6	1
*TP53*	*PTEN*	319	29	56	8	1
*IDH1*	*RB1*	379	13	18	2	1
*NRAS*	*IDH1*	283	11	109	9	1
*TP53*	*NRAS*	254	94	40	24	1
*BRAF*	*MAP2K1*	201	7	189	15	1
*TP53*	*NF1*	301	47	49	15	1
*BRAF*	*PTEN*	201	7	174	30	1

**Table 3:** Testing for mutually exclusive mutation patterns found in cell lines (*n* = 53) by the Comet Exact Test with multiple testing correction by the Bonferroni method

Gene 1	Gene 2	NeitherMutated	Gene 2Mutated	Gene 1Mutated	Both Mutated	*p*-value
BRAF	NRAS	12	5	34	2	0.438538739
BRAF	MAP2K1	17	34	2	0	1
BRAF	IDH1	17	35	1	0	1
BRAF	RB1	17	35	1	0	1
BRAF	NF1	14	3	33	3	1
TP53	NF1	30	5	17	1	1
TP53	PTEN	30	5	17	1	1
TP53	MAP2K1	33	2	18	0	1
BRAF	TP53	10	7	25	11	1
NF1	PTEN	41	6	6	0	1
TP53	IDH1	34	1	18	0	1
TP53	RB1	34	1	18	0	1
NF1	MAP2K1	45	2	6	0	1
NRAS	IDH1	45	1	7	0	1
NRAS	RB1	45	1	7	0	1
NF1	IDH1	46	1	6	0	1
NF1	RB1	46	1	6	0	1
PTEN	IDH1	46	1	6	0	1
PTEN	RB1	47	5	1	0	1
MAP2K1	IDH1	50	1	2	0	1
MAP2K1	RB1	51	1	1	0	1
IDH1	RB1	51	1	1	0	1
NRAS	NF1	41	5	6	1	1
NRAS	PTEN	41	5	6	1	1
TP53	NRAS	31	4	15	3	1
BRAF	PTEN	16	1	31	5	1
NRAS	MAP2K1	45	1	6	1	1
PTEN	MAP2K1	46	1	5	1	1

**Figure 6:**
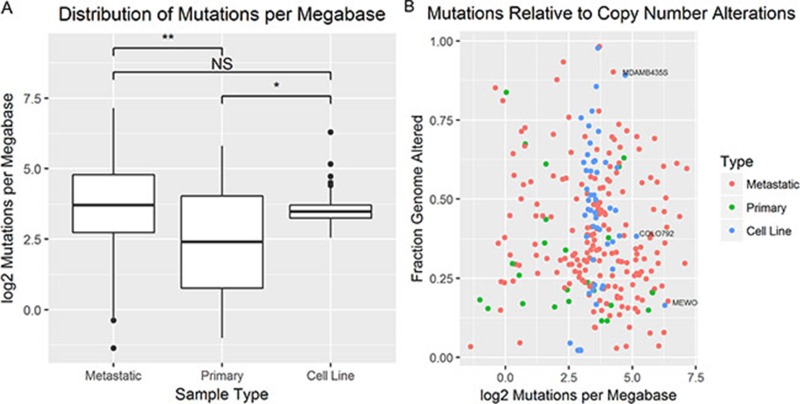
The mutational burden was compared between tumors and cell lines (**A**) Boxplots compare the distribution of log2 normalized mutations per megabase between primary tumors, metastatic tumors, and cell lines (^*^p-value < 0.05, ^**^p-value < 0.01 by ANOVA and Tukey’s Method). (**B**) A scatter plot of the fraction genome altered relative (x-axis) to the log2 normalized mutations per megabase (y-axis) for primary tumors (green), metastatic tumors (red), and cell lines (blue).

In order to compare relative total mutations between samples, mutations per megabase were calculated as described in the methods section. The mean log2 normalized mutations per megabase between metastatic tumors (*n* = 189), primary tumors (*n* = 24), and cell lines (*n* = 53) were found to be significantly different in at least two groups (*p* ≤ 0.05 by one-way ANOVA) (Figure 6A). Pairwise comparisons of mean log2 normalized mutations per megabase were carried out by Tukey’s Method. Mean log2 normalized mutations per megabase were found to be significantly higher in cell lines than primary tumors (*p* < 0.05), not significantly different between cell lines and metastatic tumors (*p* = 0.99), and significantly higher in metastatic tumors than primary tumors (*p* < 0.05). Though the mutational load of primary tumors was found to be significantly different from metastatic tumors and cell lines, sequencing coverage information was only available for a small subset of primary tumors. Since the genome size may affect the total number of mutations present, log2 normalized mutations per megabase were plotted against FGA for each cell line that was profiled for copy number alterations and DNA sequence (*n* = 52) and tumor samples (*n* = 213) (Figure 6B). The distributions are largely overlapping with all cell lines falling within the metastatic tumor distribution. There appears to be no linear association between FGA and log2 normalized mutations per megabase. The three cell lines with the highest mutational burden include MEWO, MDAMB435S, and COLO849. Of these three cell lines, MEWO has the highest mutational burden relative to its FGA.

UV radiation induced mutation signature is a common genomic feature of skin cancers like melanoma and has been previously been defined by C> T transitions at dipyrimidine sites making up ≥ 60% of all mutations or CC>TT making up ≥5% of all mutations [19, 28]. Of the filtered tumor samples, 65.3% (269/412) harbor a UV mutation signature. Of the cell lines with DNA sequencing data, 15.1% (8/53) harbor a UV mutation signature including CHL1, G361, SKMEL30, COLO792, WM88, IPC298, SKMEL5, and HS934T.

## DISCUSSION

In this study, commercially available melanoma cell lines profiled by CCLE were compared to tumors profiled by TCGA-SKCM according to genomic features including the number of mutations per megabase, the presence of differentially mutated genes, the presence of mutually exclusive mutations, total copy number alterations in the form of FGA, and the correlation of copy number alterations per gene.

**Figure 7:**
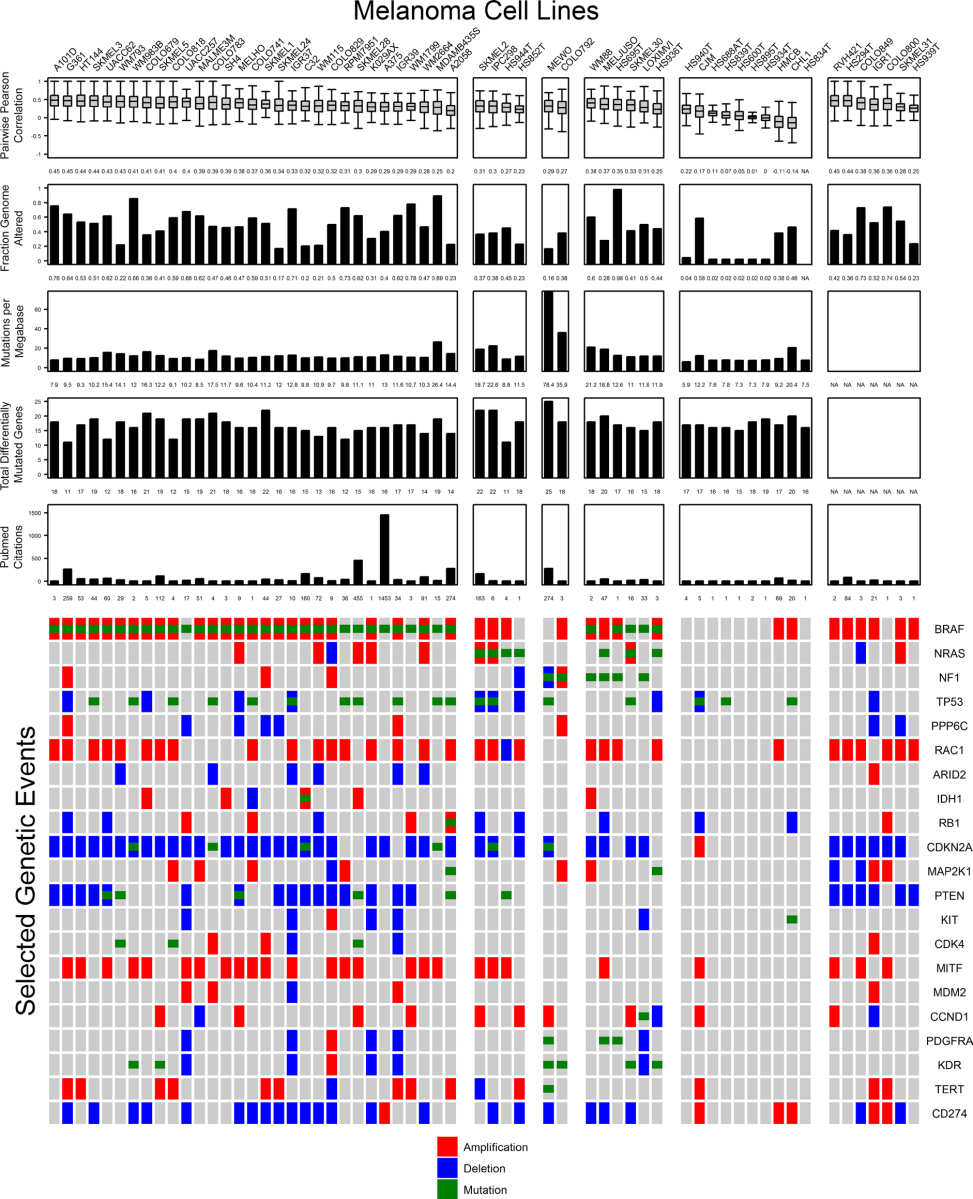
Important genomic features of melanoma cell lines relative to tumor samples are summarized with representative figures of comparisons to tumors Cell lines (columns) are grouped according to their mutation status in the top three mutated genes: *BRAF, NRAS*, or *NF1.* Cell line summary features from top to bottom include the distribution of Pearson r values between cell lines and tumors for focally amplified and deleted genes by GISTIC 2.0, the fraction genome altered, total mutations per megabase, the number of differentially mutated genes present, and the number of PubMed abstracts that include the name of the cell line. Genes included in the original TCGA-SKCM study that were significantly mutated, significantly amplified, or significantly deleted are illustrated on the bottom of the figure. Amplifications (red) include ≥ 2.5 total copies, deletions (blue) include ≤ 1.5 total copies, and mutations (green) include insertions, deletions, or single nucleotide polymorphisms.

Based on these criteria, each melanoma cell line can be evaluated by its degree of similarity to a large sample of highly annotated tumors from TCGA-SKCM. Selecting a cell line model for studying cancer biology depends on multiple factors. For melanoma cell lines, the preeminent factors are the presence of the most significantly mutated genes (*BRAF*, *NRAS*, *NF1*, or triple wild type) and proliferative or invasive behavior (*MITF*/*AXL* expression ratio). However, proliferative and invasive phenotypes are not a characteristic of all melanoma cells within a single tumor, and a spectrum of *MITF* expressing and *AXL* expressing cells exist in any single tumor that can be manipulated through treatment with RAF and MEK inhibitors [29]. Cell lines expressing predominantly *MITF* become a hierarchically organized mass of *MITF* high and *MITF* low expressing cells after growth in a mouse xenograft [30]. Hypoxia was found to alter the expression of *MITF* via HIF1α leading to switching of melanoma proliferative to invasive phenotype [31]. One mechanism maintaining the proliferative and invasive phenotypes in cell culture is through *SOX9* promoter methylation which leads to the proliferative phenotype by expression, and the overexpression of *SOX9* promotes the invasive phenotype in a mouse model [32]. Since both chemical treatment and environmental conditions may manipulate the expression signatures of melanoma cells, genomic characterization of cell lines may offer a more stable metric of cell line suitability for modeling cancer biology. For this reason, cell lines are summarized by their mutational subtype (Figure 7). The most appropriate cell line models can be selected according to the features of interest found in cutaneous melanoma. Figure 7 summarizes the major genomic features investigated in this study and provides a tool for selection of cell lines based on genetic criteria. Cell lines are organized into mutational subtypes ordered from left to right according to highest to lowest mean correlation coefficient by copy number per gene for GISTIC peak amplified and deleted genes. Copy number data was available for all cell lines except HS834T. Cell lines with strongly positive Pearson r values to a large proportion of tumors are available for each *BRAF, NRAS*, and *NF1* subtypes. MEWO and COLO792 were the only two *NF1* mutants, and both have much larger numbers of mutations per megabase relative to other cell lines. However, the mutational burden for both falls within the distribution of tumor mutations per megabase.

Both Pollock et al. and the original TCGA-SKCM study found *BRAF* and *NRAS* were anti-correlated [19, 33]. In this study using the subset of TCGA-SKCM samples filtered by estimated tumor purity, *BRAF* and *NRAS* mutations were found to occur in a mutually exclusive pattern. Additionally, *BRAF* and *NF1* mutations as well as *NRAS* and *PTEN* mutations were found occur in a mutually exclusive pattern, but none of these gene pairs were mutually exclusive in cell lines. The occurrence of *NRAS* and *PTEN* mutations were previously found to occur rarely in cutaneous melanoma [34]. *NF1* mutations generally occur with wild type *BRAF* and *NRAS* [35]. WM88, HS695T, SKMEL30, LOXIMVI, HS936T, and HS944T all harbor one of these mutually exclusive mutation pairs. These cell lines are less likely to represent broadly relevant models of cutaneous melanoma. However, activating mutations in NRAS have been proposed as a mechanism of RAF inhibitor resistance potentially making SKMEL30 or HS936T potential models for primary resistance according to this mechanism [36]. MELJUSO harbors mutations in both *NF1* and *NRAS*, but these were not found to be mutually exclusive in tumors. Vincent & Postovit found *TP53* to be mutated in a significantly higher proportion of cell lines profiled by Klijn et al. than TCGA-SKCM tumors [13, 37]. In the subset of TCGA-SKCM tumors compared in this study, *TP53* was not found to be mutated in a significantly greater proportion of cell lines (18/53) than tumors (64/412) (Bonferroni adjusted *p*-value ≥ 0.05 by Fisher’s Exact Test). The result may be dependent on different coverage requirements for mutation calls, the number of genes compared, and the different sample sets used.

Tumors that lack mutations in *BRAF, NRAS*, and *NF1* were defined in the TCGA-SKCM study as triple wild type. The triple wild type melanoma cell lines compared in this study have poor correlation with tumors by copy number per gene with mean Pearson r values between 0.22 for HS940T and −0.14 for CHL1. Uncharacterized early passage lines made available by ATCC including HS839T, HS600T, HS934T, HS895T, HS940T, and HS688AT had different molecular characteristics relative to tumors and other cell lines. All six of these cell lines had very low correlation of copy number per gene for focal amplifications and deletions identified by GISTIC 2.0 analysis and FGA near zero. Mutations per megabase in these samples were also lower than other cell lines (mean = 4.4 mutations per megabase). One potential reason for the lack of similarity may be due to a relatively high concentration of stromal cells relative to tumor cell lines in these early passage cell cultures. ESTIMATE scoring indicated that these cell lines had elevated stromal scores relative to other cell lines. CJM, HMCB, and CHL1 also lacked mutations in *BRAF*, *NRAS*, or *NF1*, but these cell lines had similar FGA and mutational burden relative to other melanoma cell lines as well as tumors. However, these three cell lines also had very low mean correlation to tumors by copy number alterations in focally amplified and deleted genes. HMCB and CHL1 were annotated as having high SNP similarity in the original CCLE study [16]. Both HMCB and CHL1 show very poor correlation with copy number per gene in focally amplified and deleted genes with mean correlation coefficients of −0.11 and −0.14 respectively, but these two cell lines had the most citations relative to other triple wild type cell lines. There is an overall lack of studies including triple wild type melanoma cell lines, and the commercially available cell lines compared in this study have low similarity to melanoma tumors relative to other cell lines, indicating new cell lines may be warranted.

This study has identified features of melanoma cell lines which may indicate that they are not accurate models of most human melanomas. Though a UV mutation signature is common in melanoma and occurs in 65.3% (269/412) of TCGA-SKCM tumors, only 15.1% (8/53) cell lines harbor a UV signature. The UV signature may be lost during maintenance of cell lines as more mutations are acquired making the UV signature less discriminatory and more descriptive of a cell line’s origin. Most cell lines have good correlation of DNA copy number per gene for focally amplified and deleted genes by GISTIC 2.0 analysis, but a subset of tumors was found to have low similarity across cell lines independently of tumor stage, primary or metastatic status. Cell lines which harbor mutations of interest, the fewest differentially mutated genes, and the highest Pearson sample correlation with most tumors provides criteria to select cell lines with more genetic similarity to patient tumors.

There are several limitations to conclusions drawn from this study. DNA sequencing data from CCLE was only available for a set of 1651 genes selected for hybrid capture sequencing. Specific mutations, mutational burden, and UV signature were drawn only from this small subset of genomic data. Additionally, all data types were not available for all cell lines, limiting the full characterization of each one. Despite these limitations, comparison of cell line molecular features may lead to more applicable pre-clinical models that can improve the translational efficiency of *in vitro* studies.

## MATERIALS AND METHODS

### Data sets

All files were downloaded in March of 2017. Cell line DNA copy number, DNA mutation, and mRNA expression data were obtained from the Broad Institute data portal (https://portals.broadinstitute.org/ccle/data/) as described in the original CCLE study [16]. Normalized segmented DNA copy number data obtained by Affymetrix SNP 6.0 array was downloaded as CCLE_copynumber_2013-12-03.seg.txt, which includes 59 melanoma cell lines. DNA mutation data was obtained with the Agilent Sure-Select Target Enrichment System including 1651 selected genes downloaded as CCLE_hybrid_capture1650_hg19_NoCommonSNPs_NoNeutralVariants_CDS_2012.05.07.maf, which includes 53 melanoma cell lines. The CCLE MAF file included only coding regions, excluded common polymorphisms, mutations with a variant allele frequency < 10%, and putative neutral variants. Sequencing coverage for all 53 CCLE samples included in the MAF file was provided in WIG format and downloaded as CCLE_hybrid_capture1650_hg19_coverage_2012.06.19.tar.gz. Affymetrix U133+2 array mRNA expression data was obtained as normalized gene level data from CCLE_Expression_Entrez_2012-10-18.res. All the CCLE data was mapped using the hg19 genome build.

TCGA-SKCM melanoma tumor data from 470 cases including DNA copy number, DNA mutation, and mRNA expression were downloaded with the TCGAbiolinks R package from the GDC legacy archive (https://portal.gdc.cancer.gov/legacy-archive/) mapped to the hg19 genome to facilitate comparison with CCLE [19, 38]. Multiple samples from single cases were available for some data platforms, and the total number of samples is noted for each data type. Normalized segmented DNA copy number data obtained by Affymetrix SNP 6.0 was downloaded as 471 SEG files. Somatic DNA mutation data was downloaded as a single MAF file, SKCM_pairs.aggregated.capture.tcga.uuid.automated.somatic.maf. Since the sequencing coverage for TCGA is not made publicly available, sequencing coverage for TCGA-SKCM data was downloaded from Synapse (https://www.synapse.org/) via syn1709990 as 255 BED files containing genome regions covered with at least 14 reads (*n* = 255) as described by Kandoth et al. [39]. Illumina Hiseq mRNA expression data were downloaded as 472 RSEM normalized results files.

### Tumor cell fraction by ESTIMATE Score

The ESTIMATE R package was used to calculate ESTIMATE scores for CCLE samples using Affymetrix U133+2 array normalized mRNA expression data and for TCGA-SKCM tumors using normalized RNA-seq data [23]. Tumor cell fraction was estimated for each TCGA-SKCM sample and all CCLE cell lines with the ESTIMATE score using the formula published by Yoshihara *et al.* [23]. Gene names were matched by HGNC symbols.

### Fraction genome altered

The fraction genome altered was calculated using the formula published by Domcke *et al* with some modification to the mathematical notation:

$$\text{FGA}=\sum_{i[|{\text{CN}}_{i}|>T]}^{n}L_{i}/\sum_{i=1}^{n}L_{i}$$

where *Li* represents length *L* of each segment *i* and *CNi* represents normalized copy number *CN* for each segment *i* with a chosen normalized copy number threshold *T* for *n* total segments [10]. Therefore, FGA for a sample is the sum of all segment lengths where the absolute value of the normalized copy number value is above a chosen threshold divided by the sum of all segment lengths.

### DNA copy number correlation

A reduced segment matrix was extracted from segmented copy number data for each CCLE and TCGA-SKCM sample and was used to calculate mean copy-number per gene with CNTools [40]. The CNTools algorithm aligns segments across samples, and genes that fall within an overlapping segment are assigned the mean of the probe log2 ratios within that segment. Genome coordinates of known protein coding genes or known non-coding RNAs were downloaded with biomaRt for the hg19 ENSEMBL genome build including 23,959 genes [41, 42]. Genes on X and Y chromosomes were omitted to facilitate comparison between male and female derived samples resulting in 22,780 genes with mean copy number values for each sample. Pearson sample correlation coefficients of copy number per gene were calculated between each cell line and each tumor individually as a correlation matrix to obtain the distribution of copy number per gene similarity. Additionally, the mean was calculated for copy number values for each gene across all TCGA-SKCM samples. Pearson sample correlation coefficients of copy number per gene were calculated between each cell line and the mean of all tumors to assess which cell line shows the most genomic similarity to tumor samples as a group. Finally, cell lines and tumors were compared by average copy number per gene for genes within the peak of significant focal amplifications and deletions found in metastatic tumors from TCGA-SKCM (*n* = 367) by GISTIC 2.0 analysis downloaded from the Broad Institute GDAC Firehose [26].

### Comparing mutations

Differentially mutated genes were identified using Fisher’s Exact Test implemented via maftools to compare the proportion of tumors relative to the proportion of cell lines that carry a mutation in a given gene with a minimum of five samples carrying the mutation [43]. Comparisons were limited to 1,192 genes that were mutated in both the TCGA-SKCM MAF file and the CCLE MAF file with variant allelic fraction ≥ 0.1 and ≥ 8 reads total per variant. Synonymous variants were excluded from comparison. Eight significantly mutated genes reported in the original TCGA-SKCM study were present in the cell line MAF file including *BRAF*, *TP53*, *NF1*, *NRAS*, *PTEN*, *MAP2K1*, *IDH1*, and *RB1*. These genes were tested for the occurrence of mutations in a mutually exclusive pattern each in tumors and cell lines using cometExactTest via maftools [27]. Multiple testing corrections were implemented for both Fisher’s Exact Test and the Comet Exact Test by the Bonferroni method implemented through p.adjust in the R programming environment.

### Comparing the number of mutations normalized by coverage

In order to compare mutations between CCLE data and TCGA-SKCM data, the total number of mutations was normalized by the breadth and depth of coverage for each data set. Sequencing coverage data was available for 255 melanoma tumor samples provided from the Synapse data sharing platform, and 213 of those tumor samples had tumor cell fraction greater than 60% calculated using ESTIMATE scores. The BED files contain genomic start and end positions for regions covered by ≥14 reads. The breadth of sequencing coverage for TCGA-SKCM samples was calculated by subtracting each end position from each start position in the BED file, and total breadth of coverage was calculated by summing all lengths. Coverage was available for CCLE samples as WIG files, which contain the number of reads covering each genome position. In order to have equivalent breadth of coverage for both CCLE and TCGA-SKCM samples, the total length covered for CCLE samples was calculated by counting the total positions provided in the WIG files with ≥14 reads. Total mutations were counted for TCGA-SKCM and CCLE from their respective MAF files filtered by variant allelic fraction ≥ 0.1 and ≥ 14 reads including synonymous variants, insertions, deletions, and single nucleotide polymorphisms. Total coverage-normalized mutations were calculated by dividing the sum of all coding region mutations from the MAF files divided by the sequencing coverage calculated for each of the TCGA-SKCM samples and CCLE samples.

### UV Signature

UV signature for TCGA-SKCM samples (*n* = 412) and CCLE samples (*n* = 53) was determined by extracting the flanking bases surrounding mutations from MAF files using the maftools and summing the number of C>T transition mutations flanked by either C or T; then dividing the sum by the total number of substitution mutations in the MAF file. MAF files for TCGA-SKCM and CCLE were filtered by variant allelic fraction ≥ 0.1 and ≥ 8 reads including synonymous variants, insertions, deletions, and single nucleotide polymorphisms. A UV signature was defined as C>T transitions occurring at dipyrimidine sites comprising ≥ 60% of all substitution mutations or ≥5% CC>TT mutations.

### Software

Data processing and statistical analysis was carried out using Linux shell scripting and R version 3.3.2 [44]. Bioconductor packages were applied as necessary including CNTools, biomaRt, TCGAbiolinks, ComplexHeatmap, maftools, cometExactTest and RColorBrewer [45]. Other R packages were applied to create plots and make calculations including dplyr, ggplot2, reshape2, and ggsignif. Hierarchical clustering for all heat maps was carried out using Euclidean distance and Ward’s agglomeration method.

### PubMed citations

PubMed titles and abstracts (https://www.ncbi.nlm.nih.gov/pubmed) were searched on June 7^th^, 2017 using the advanced search builder with multiple punctuation combinations including spaces, hyphens, and periods. Additionally, the search term “melanoma” was required in combination with each cell line name to avoid false-positive search hits. For cell lines with zero search hits, the number of cell line studies annotated by Cellosaurus (web.expasy.org/cellosaurus) was summed to account for publications which don't have the cell line names included in the title or abstract [46].

## Abbreviations

CCLE: Cancer Cell Line Encyclopedia
CNA: copy number alteration
ESTIMATE: Estimation of Stromal and Immune cells in Malignant Tumor tissues using Expression data
FGA: fraction genome altered
TCGA-SKCM: The Cancer Genome Atlas Skin Cutaneous Melanoma
